# Cancer Stem Cells: From an Insight into the Basics to Recent Advances and Therapeutic Targeting

**DOI:** 10.1155/2022/9653244

**Published:** 2022-06-28

**Authors:** Shweta Bisht, Manisha Nigam, Shyam S. Kunjwal, Plygun Sergey, Abhay Prakash Mishra, Javad Sharifi-Rad

**Affiliations:** ^1^Department of Biochemistry, Hemvati Nandan Bahuguna Garhwal University, Srinagar Garhwal, 246 174 Uttarakhand, India; ^2^Department of Zoology, School of Sciences, Uttarakhand Open University, Haldwani, 263139 Uttarakhand, India; ^3^European Society of Clinical Microbiology and Infectious Diseases, Basel 4051, Switzerland; ^4^Laboratory of Biocontrol and Antimicrobial Resistance, Orel State University Named after I.S. Turgenev, 302026 Orel, Russia; ^5^Department of Pharmacology, Faculty of Health Science, University of Free State, Bloemfontein 9300, South Africa; ^6^Facultad de Medicina, Universidad del Azuay, Cuenca, Ecuador

## Abstract

Cancer is characterized by an abnormal growth of the cells in an uncontrolled manner. These cells have the potential to invade and can eventually turn into malignancy, leading to highly fatal forms of tumor. Small subpopulations of cancer cells that are long-lived with the potential of excessive self-renewal and tumor formation are called cancer stem cells (CSCs) or cancer-initiating cells or tumor stem cells. CSCs can be found in tissues, such as breast, brain, lung, liver, ovary, and testis; however, their origin is still a matter of debate. These cells can differentiate and possess self-renewal capacity maintained by numerous intracellular signal transduction pathways, such as the Wnt/*β*-catenin signaling, Notch signaling, transforming growth factor-*β* signaling, and Hedgehog signaling. They can also contribute to numerous malignancies and are an important reason for tumor recurrence and metastasis because they are resistant to the known therapeutic strategies that mainly target the bulk of the tumor cells. This review contains collected and compiled information after analyzing published works of the last three decades. The goal was to gather information of recent breakthroughs related to CSCs, strategies to target CSCs' niche (e.g., nanotechnology with tumor biology), and their signaling pathways for cancer therapy. Moreover, the role of metformin, an antidiabetic drug, acting as a chemotherapeutic agent on CSCs by inhibiting cellular transformation and its selective killing is also addressed.

## 1. Introduction

Cancer stem cells (CSCs) represent specific type of rare cells found in the broad majority of tumors, possessing self-renewal and differentiation capacities. They contribute to the heterogeneous lineages of the cancer cells that form the tumor and share some of the common characteristics with stem cells. The concept of CSCs has been traced back to early 1900s when Julius Friedrich Cohnheim observed the similarity of the tissues of teratocarcinomas with the embryonic tissue. Cohnheim supported the theory of “embryonic rests” using the resemblance. In 1964, G. Barry Pierce showed that embryonal carcinoma (EC) cells that are derived from embryonal teratocarcinoma and carcinomas could generate multiple differentiated tissues and cause embryonal carcinoma upon transplantation into the mice. After this finding, he interpreted his observation supporting the theory of CSCs and stated that EC cells are multipotent. In the 1980s, the impacts of oncogenes on proliferation and genomic stability were given more attention than tumor differentiation issues. It continued till 1990s, because many researchers considered teratocarcinomas, which provided as a model for investigating differentiation phenomenon, as a unique case with little importance to the study of other cancers. The study of acute myelogenous leukemia (AML) led to the increased interest to explore more about the concept of CSCs [1].

In 1997, Dominique Bonnet and John E. Dick [2] were the first to identify CSCs from the mononuclear cells of the blood of human acute myeloid leukemia (also known as AML). They explained that these cells share similar characteristics (the ability of self-renewal and cellular differentiation into another cell type) with the normal stem cells (NSCs) and possess an exclusive phenotype of CD34^+^ and CD38^−^ surface markers and can be differentiated into leukemic blasts [2]. However, normal stem cells are noticeable for the diligence with which they control their proliferation and the ability with which they maintain their genomic integrity. Three distinctive properties of CSCs (self-renewal, the ability to develop into multiple lineages, and the potential to multiply quickly) are somewhat related to various forms of cancer [3]. CSCs also possess the property of plasticity via reversibly switching between the stem and nonstem cell states. They can escape apoptosis and can metastasize, though they may remain dormant for long duration. CSCs have the potential for self-renewal, can develop to all types, and are found in a specific sample of cancer having the capacity of continuous expansion into the population of malignant cells [2, 4, 5]. Due to their ability of tumor initiation, these cells are believed to play a crucial role in tumorigenic processes, such as oncogenesis, metastasis, and cancer recurrence [6]. Tumorigenesis and the behavior of the cell depend upon the microenvironment of tumor, and their identification depends on the surface markers along with their potency of self-renewal and propagation. CSCs contribute to the tumor heterogeneity, managing the vital malignant behaviors of processes such as invasion, metastasis, and therapy resistance, which is caused by epigenetic and genetic pathways. Many intracellular as well as extracellular factors that can be used for targeting drugs to treat cancers are responsible for controlling the activities of CSCs [7].

CSCs do not easily get destroyed via chemotherapy and radiotherapy, which means that following the effective destruction of the bulk of tumor by various therapies, a subset of residual CSCs may survive and facilitate cancer relapse leading to invasiveness and therapy resistance, thereby playing a critical role in the progression of cancer and therapy resistance. To prevent this condition, a deep understanding of the biology of CSCs is essential to develop effective therapies. Various CSC-targeted specific therapies are developing across the globe, for improving the survival rate and quality life of cancer patients, specifically for those with metastatic disease [8].

This review includes findings on CSCs from the recent reviews and research papers searched on PubMed database, using keywords such as “cancer,” “self-renewal,” “stemness,” “tumorigenesis,” and “signaling.” The goal was to summarize a detailed and conclusive understanding of the biological characteristic properties of CSCs, hypotheses of their origin, identification and regulating signaling pathways. Furthermore, recent breakthroughs in therapeutic strategies to target CSCs for cancer therapy are also discussed, with a focus on the role of metformin, an antidiabetic drug that acts as a chemotherapeutic agent against CSCs. Nevertheless, combining nanotechnology with tumor biology is considered to be critical, since nanosized materials may be exploited for CSC-driven anticancer therapy.

## 2. Theories of Origin and Niches of Cancer Stem Cells

### 2.1. Theories of Origin

**Figure 1:**
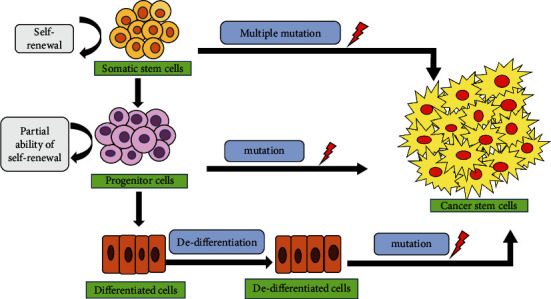
Theories of origin of cancer stem cells. There are three possible theories: (i) CSCs could have possibly originated either from normal stem cells when they underwent mutation or oncogenic transformation, (ii) from progenitor cells who also undergone mutations, and (iii) from the fully differentiated cells that undergone several mutations via dedifferentiation (curved black arrows indicate self-renewal, while the straight arrows indicate the expression promotion).

The theory of cancer stem cells states that a given subgroup of cancer cells drives tumor spread and growth which causes the progeny of cancer cells to be highly differentiated and destined to cease the proliferation because they have restricted mitotic divisions. The theory of CSCs (Figure 1), therefore, shows that certain features of the cellular hierarchy which are observed in normal tissues can be seen in many tumors (Table 1 and Table 2). It was earlier suggested that CSCs are born from NSCs (Table 3), by observing the capability of differentiation of the leukemic cells into multiple mature lineages and allocating the expression of few markers with the NSCs [4].

Based upon the application of stem cell concepts, which are known to be derived from embryogenesis to understand the process of tumorigenesis, some of the key features of CSC hypothesis are as follows: (i) When cancer cells were transplanted into immunodeficient mice, the cancer cells having tumorigenic potential were present in a small fraction [7]. (ii) By using distinctive surface markers, the CSC subpopulation can be distinguished from the other cancer cells [7]. (iii) Tumorigenic and nontumorigenic cells of the original tumor are present as a mixture in the tumor which is the result of CSCs [7]. (iv) The subpopulation of CSCs can be transplanted in series through several generations, indicating that it is a self-renewing population [7].

The progenitor cells arise from various types of stem cells possess the ability to divide further into differentiated or specialized cells for performing specific functions of the body. The origin of CSCs is still a controversy that whether they arise from stem cells, progenitor cells, or differentiated cells those present in adult tissues [13].

### 2.2. Niches of Cancer Stem Cells

Similar to normal stem cells, CSCs also reside in niches which are the specialized microenvironment known for regulating the normal stem cell fate via providing signals either by some secreted factors or through cell-cell contacts. Niches for mammalian stem cells have been identified in various epithelial tissues, such as the intestine, neural, epidermal, and hematopoietic systems. The components of normal niches are fibroblastic, endothelial, and perivascular cells or their progenitors, immune cells, extracellular matrix (ECM) components, networks of cytokines, and growth factors [12].

**Figure 2:**
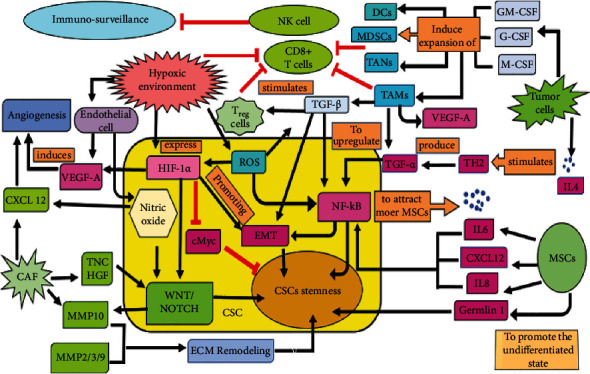
Crosstalk between CSCs and their niches. Cells present in the CSC niche produce some factors that stimulate self-renewal and angiogenesis and secrete factors involved in tumor cell invasion and metastasis. MSCs secrete CXCL12, IL-6, and IL-8 (the black arrows indicate the expression promotion) which promote CSC stemness via upregulating NF-*κ*B. To attract more MSCs towards CSCs, the latter also secretes IL-6. Gremlin 1 is an antagonist produced by MSCs to boost up the undifferentiated state. The tumor cells present around the CSC produce IL-4 which stimulates T_H_^2^ and further produce TNF-*α* for upregulating the NF-*κ*B signaling. GM-CSF, G-CSF, and M-CSF are also produced by the same tumor cell to induce the expansion of some immune cells such as TAMs, TANs, MDSCs, and DCs. To enhance the plasticity of CSCs, TNF-*α* and TGF-*β* are produced by TAM to promote the NF-*κ*B-dependent or TGF-*β*-dependent EMT. TGF-*β* is also being produced by TAMs to stimulate the T_reg_ cells. TAM, T_reg_, and hypoxic microenvironment also inhibit CD8^+^ T cell, NK cell cytotoxicity, and phagocytosis of macrophages thus inhibiting immunosurveillance (red arrows depicting inhibition). Hypoxic microenvironment increases the concentration of ROS, promotes cell survival, and induces EMT via the TGF-*β* signaling pathway. The downregulated c-Myc expression inhibits cell proliferation under hypoxia and enhances stemness. CXCL12 is produced by CAF to promote angiogenesis. Under hypoxic microenvironment, CSCs and ECs produce VEGF, which further induces angiogenesis. Nitric oxide production via the Notch signaling pathways leads to the self-renewal of CSCs. CAFs also produce TNC, HGF, and MMP2/3/9, which help in the enhancement of the Wnt and Notch signaling. It also produces MMP10 which promotes ECM degradation and remodeling thus enhances the CSC's stemness.

Cancer consists of malignant cells along with inflammatory cells, associated hematopoietic cells, stroma, and vasculature. So, the effect of niche may be inductive or selective depending upon subtype of every tumor. In the case of glioblastomas, there is a bidirectional relationship between the CSCs and the local environment as the niche can alter the cellular fate of cancer cells and can modify their microenvironment [14]. The CSC niche itself is isolated from the tumor microenvironment (TME), which is a collective term for neighboring stroma together with normal counterparts of tumorigenic cells [15]. The cells present in CSC niche produce some factors which further help in stimulating the self-renewal property of CSCs and induce angiogenesis (Figure 2) [15]. Some additional factors, secreted by immune cells and other stromal cells that are recruited by cells residing in CSC niches, promote tumor cell invasion and metastasis via transdifferentiation into the vascular cells. CSCs which are present in glioblastomas contribute to the microvasculature [14], highlighting the close relationship between brain CSCs and their niche. In the case of cutaneous squamous cell carcinomas, the perivascular niche plays a very crucial role. Signals are provided by cellular and noncellular components of the niche which in turn regulate the proliferative and self-renewal signals in order to help CSCs so that they can maintain their quiescent state [15].

Mesenchymal stem cells (MSCs), being the multipotent stromal cells, secrete CXCL12, interleukin-6 (IL-6), and IL-8 which promote CSC stemness via upregulating NF-*κ*B. Gremlin 1, an antagonist, is also produced by MSCs to boost up the undifferentiated state [15].

The tumor cells surrounding CSC produce IL-4, which stimulates T_H_^2^ to further produce TNF-*α* for upregulating the NF-*κ*B signaling pathway. Along with IL-6, granulocyte-macrophage colony-stimulating factor (GM-CSF), G-CSF, and M-CSF are also produced by the same tumor cell for the expansion of some immune cells such as tumor-associated macrophages (TAMs), tumor-associated neutrophils (TANs), myeloid-derived suppressor cells (MDSCs), and dendritic cells (DCs). T regulatory cells (T_reg_) participate in immunosuppression recruited by TGF-*β*, which is secreted by TAMs [15].

Myofibroblast cells present in the tumor-associated stroma secrete HGF, which helps to maintain the function of CSCs by activating the Wnt pathway in the colorectal cancer, and they are present adjacent to stromal myofibroblasts thus induce some of the CSC features and tumorigenic capacity in differentiated cancer cells having the limited tumorigenic capacity [15]. Endothelial cells in the reverse direction secrete nitric oxide which results in the induction of the Notch signaling in glioma cells. Providing nutrients and oxygen to the cells, endothelial cells also secrete some factors that play a crucial role in promoting the self-renewal property as well as help in the survival of the head and neck CSCs [13].

Many cells surrounding the CSCs such as MSCs, cancer-associated fibroblasts (CAFs), TAMs, and some nonstem cancer cells also play a very important role in maintaining the CSC stemness.

#### 2.2.1. Role of Exosomes in Tumor Microenvoirnment (TME) as well as in CSCs

Exosomes are the membrane-bound extracellular nanovesicles that are derived from the endosomes and possess a large number of functional proteins, RNA, microRNAs, and some DNA fragments [16]. They can be found in peripheral blood, breast milk, saliva, urine, and in many other body fluids. In comparison to normal cells, tumor cells secrete 10 times more vesicles, which is the most effective route for the tumor and metastatic information to reach both normal and tumor cells, as the transfer of tumor derived exosome (TDE) contents to nonmalignant cells has been shown to activate the tumor phenotype and metastatic properties [17]. The major role of exosomes when secreted in high concentration from the cancer cell is to induce differentiation of tumor-related fibroblasts, to promote angiogenesis, to regulate the microenvironment before metastasis, and to participate in the immune regulation of TME [16].

The interaction between CSCs and NSCs can be mediated by the exosome signaling which further regulates the development of tumors as well as the process of oncogenesis. By targeting some specific signaling pathways (such as Wnt, Notch, Hh, and NF-*κ*B), exosomes can regulate the growth of CSCs [18]. In the case of colorectal cancer, exosomes derived from fibroblast provide chemoresistance by promoting the growth of CSCs, while exosomes derived from CAF promote sphere formation by activating the Wnt pathway, thus increasing the number of CSCs [19]. Exosomes, when released from CSCs, increase the stemness of breast cancer cells and also affect the signal transduction in nearby cells [20]. They are considered as an ideal drug carrier for cancer therapy because of their easy storage, high drug loading capacity, long life, easy production, biocompatibility, and low immunogenicity [17, 18].

#### 2.2.2. Role of Hypoxic Microenvironment on Cancer Stem Cells

Hypoxia is an oxygen-deprived condition of the body resulting in the insufficient supply of the oxygen at the tissue level. This deprived state can promote genetic instability, metastasis, and invasiveness of tumor cells, resulting in the expression of HIFs by CSCs, where TGF-*β* is responsible for their regulation and stabilization. In order to adapt in this state, there is a phenotypic shift in the expression of genes that regulates the various cellular processes [21].

**Figure 3:**
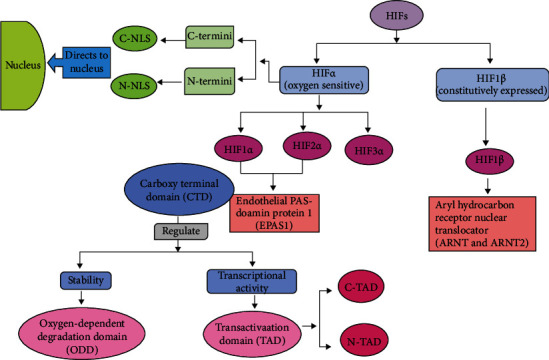
Structural and functional organization of hypoxic inducible factors (HIFs). HIF is a heterodimeric complex composed of an oxygen-dependent *α*-subunit (HIF-*α*) and an oxygen-insensitive *β*-subunit (HIF-*β*). The HIF-*α* has three subunits (HIF-1*α*, HIF-2*α*, and HIF-3*α*). The regulation of HIF-1*α* and HIF-2*α* is done by oxygen tension and is ubiquitously expressed in the normal tissue, whereas the HIF-1*β* is the subunit of HIF-*β*. The carboxy-terminal domain (CTD) of HIF-1*α* and HIF-2*α* based on their regulation is divided (indicated by black arrows) into two domains: ODD (regulates stability) and TAD (regulates transcriptional activity via two transactivation domains (TADs))—(i) N-TAD and (ii) C-TAD. Some nuclear localization signals (NLS) are present in both the C and N-termini of the *α*-subunits such as N-NLS and C-NLS that give it the direction towards the nucleus.

**Figure 4:**
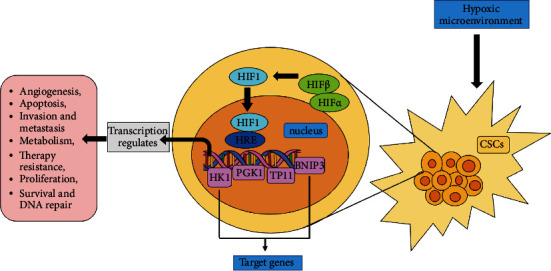
Role of HIFs in CSCs. Under hypoxia, HIF-1 is formed after the dimerization of HIF-1*α* with HIF-1*β* and binds to the HRE (HIF-responsive element) present at the DNA. This binding results in the transcription of the targeted genes (HK1, PGK1, TP11, and BNIP3) (depicted in pink boxes) and regulates the various cellular processes like survival, angiogenesis, apoptosis, invasion, metastasis, metabolism, therapeutic resistance, and DNA repair.

Hypoxia-inducible factors (HIFs), central to this shift, act as mediators under the hypoxic microenvironment for monitoring cellular responses to the oxygen level [21]. HIFs being heterodimeric are those helix-loop-helix transcription factors having an *α* and a *β* subunit (HIF-*α* and HIF-*β*), as shown in Figure 3 [22]. Under the hypoxic condition in order to self-sustain, the role of HIFs in CSCs is to promote stemness and regulate tumor growth and cell survival by activating HIF-1 and HIF-2 as summarized in Figure 4 [23]. It was concluded from the knockdown experiments performed *in vivo* that those CSCs having low HIF activity are unstable in tumor propagation and cell survival. Furthermore, hypoxia increases the expression of Sox2 and Oct4 genes, both of which are involved in stem cell function. Sox2, along with Sox4, has been revealed to play a critical role in the preservation of stemness in CSCs [22]. Notably, some genes linked with the hypoxia response in normal cells, such as Glut1, Serpin B9, and VEGF, are elevated in CSCs [21]. In the case of solid tumors, the activity of oncogene can be regulated by HIF-1*α* via various pathways such as Akt and epidermal growth factor receptor (EGFR) [21]. The specificity of HIF-*α* and HIF-2*α* is essential for the survival and propagation of tumor. HIF-1*α* when activated in hypoxia leads to the expansion of the subpopulation of the cells, which are positive for CSC marker, CD133. The level of CD44, a marker that is associated with stem-like phenotype, is also increased [21]. The expression of HIF-2*α* stimulates expression of Oct-4 and promotes the activity of c-Myc, which ultimately ensures the property of undifferentiation in CSCs [22].

Interestingly, there is a dynamic heterogeneous cellularity scattered within the edge and core of the tumor for producing adaptive mechanisms in response to various damaging situations. Edge cells are more quiescent, invasive, and resistant to infection than core cells, which multiply faster and have a higher cellular density. A rise in the number of resistant cells is an expected phenomenon in advanced malignancies and is partly due to core-to-edge cellular shifting, which expands the tumor's edge area, and hypoxia is a major contributor to this cellular dispersal [24].

The Hippo signaling pathway, in breast CSCs, is induced via HIF-1*α*, by directly targeting the Hippo pathway effector, TAZ, which is a key regulator of breast cancer stem cell (BCSC) activity [25]. In patients with glioblastoma, HIF-1*α* or HIF-2*α* are targeted in CD133^+^ cells by short hairpin RNA that inhibits its proliferation and capability of neurosphere formation and induces caspase-dependent apoptotic effect *in vivo* and *in vitro* alters their tumor-initiating potential [26]. In AML, HIF-1 is overexpressed and preferentially activated in CD34^+^ CD38 subgroups [26], which enhances the stem-like phenotype and further results in increase in the number of stem cells of leukemia. Hypoxia also regulates various signaling pathways, such as Wnt and Notch, which induce EMT, further increase the invasiveness and stemness of CSCs, and also provide resistance to radiotherapy and chemotherapy [23].

## 3. Identification of Cancer Stem Cells

CSCs, first identified in AML, were confirmed with the subpopulations of CD34^+^ and CD38^−^, similar to normal hematopoietic stem cells (HSCs) [2]. When these were transplanted into immune-deficient mice, then their tumor-initiating capacity was proved [27].

Some glycoproteins or proteinaceous markers can upregulate, downregulate, mutate, or silent the properties of CSCs. In order to detect CSCs, localization of these markers is crucial. Some of these markers are found in the cytoplasm, while others are present as the cell surface antigens [28]. In fact, breast cancer stem cells (BCSCs) were first identified in 2003 on the basis of the expression levels of cell surface antigens, CD44^+^ and CD24^−^, that account for high capacity of invasiveness, migration, and proliferation [29]. CD44, which is a transmembrane glycoprotein receptor, acts as a crucial signal molecule that interacts with the cytoskeletal proteins or modulates gene expression thus eventually alters cell behavior. It is also involved in cell adhesion and migration [28]. According to scientists, CD44 and its isoform are reliable cancer stem cell markers and can be used alone as well as in a combination with other surface markers in order to identify CSCs. ALDH1, CD133, and CD61 are the examples of other markers that are correlated with BCSCs [29].

Although the most popular method which is being used to identify specific markers for CSCs is by observing the expression of cell surface antigen, however, those markers which are used to identify stem cells from one organ are not useful for identifying stem cells in other tissues. For example, Sca-1, a marker protein used to identify murine blood stem cells, cannot consistently be expressed by murine mammary duct stem cells [30]. Different biomarkers used for the identification of CSCs in various human cancers are depicted in Table 4.

## 4. CSC Studies in Solid Tumors

An abnormal mass of tissue that does not contain any cysts or a liquid portion and can be benign (noncancerous) or malignant (cancerous) is referred to as solid tumors. The first solid tumor was studied in human breast cancer [5]. For the expression of CD44 and CD24, the samples of human breast tumors were analyzed and were found to be heterogeneous in the expression of surface antigen similar to the case of AML [27]. Human breast cancer cells were separated into different populations on the basis of the differences in the expression of surface antigen through flow cytometry. Upon injecting different populations into nonobese diabetic/severe combined immunodeficiency (NOD/SCID) mice only those cells of human breast cancer formed tumors that can express a CD44^+^ CD24^low^/^−^. Those human breast tumor cells, that are able to develop tumor when injected into NOD/SCID mice, specifically refer to “breast cancer stem cells” [92].

Using immunodeficient mice (SCID or NOD/SCID) as recipients for xenografts of the tumor, various other human solid tumors have been studied. For example, CD133^+^ and CD133^−^ were found in human brain tumors. When implanted into immunodeficient mice, only cancer cells expressing CD133^+^, named “brain tumor-initiating cells”, were capable of forming tumors in the majority of patients [27].

Flow cytometry analysis of colon cancer tissues revealed heterogeneous groups of cells inside a tumor. The recent findings showed that in many patients having human colon cancer, only the CD133^+^ subsets of tumor cells known as “colon cancer-initiating cells” were capable of creating xenografted tumors in mice [93]. In the case of human colon cancer, the tumor-forming subsets in a patient's tumor were defined using CD44, epithelial cell adhesion molecule (EpCAM also known as an epithelial-specific antigen or ESA), and CD166. Only cells that were positive for these markers were able to produce tumors in mice whether utilized in pairs or all three together (ESA^hi^CD44^+^, CD44^+^, CD166^+^, ESA^hi^CD166^+^, or ESA^hi^CD44^+^ CD166^+^) [93]. CD44^+^ plays a major role in studying head and neck squamous cell carcinomas (HNSCC). Tumorigenic cancer stem cells in HNSCC possess the ability to propagate tumor formation in mice. To study HNSCCs, both NOD/SCID and Rag2/cytokine receptor common *γ*-chain double knockout (Rag2*γ*DKO) mice were used as an immunodeficient mouse test model [92]. In human pancreatic cancers, cells having similar tumor propagation abilities were also found. They neither express CD44 nor CD24. Those cells expressing CD44^+^, CD24^+^, and ESA (named pancreatic cancer stem cells) were responsible for tumor formation [80].

## 5. Signaling Pathways Involved in CSCs

In the case of normal stem cells, various highly regulated molecular signaling pathways contribute to the different properties like self-renewal, survival, proliferation, and differentiation. On the contrary, in tumorigenesis or cancer stem cells, these signaling pathways are either repressed or abnormally activated. Moreover, these complex pathways are regulated by various extrinsic and intrinsic molecular signals, endogenous and exogenous genes, some regulatory elements, and microRNAs. Instead of being linear, these pathways are interwoven networks of signaling mediators that regulate and support the function of CSCs [19, 94]. There are nine signaling pathways that are known to be involved in embryonic development as well as in cancer so far. Out of these nine, only seven pathways (such as the JAK/STAT pathway, MAP-kinase/ErK pathway, NOTCH pathway, NF-*κ*B pathway, P13K/Akt pathway, TGF-*β* pathway, and Wnt pathway) are common in both cancer and stem cells [19].

### 5.1. Hedgehog (Hh) Signaling Pathway in CSCs

**Figure 5:**
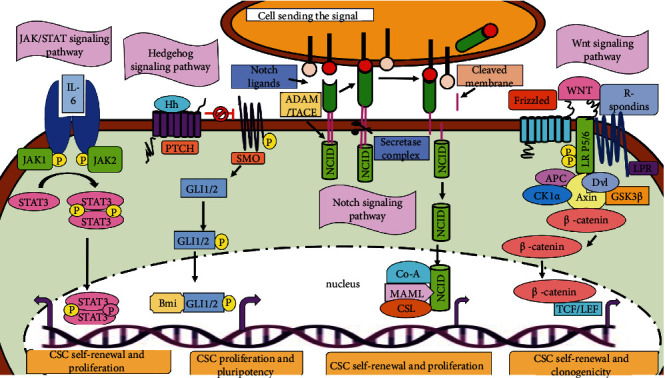
Signaling pathways involved in cancer stem cells. (a) The JAK/STAT pathway (extreme left): JAKs get activated when ligands bind to its receptor; JAK1 and JAK2 auto and transphosphorylate each other and also phosphorylate the tyrosine residues present in the cytoplasmic domain of the receptor. STATs upon phosphorylation by JAKs form dimers and are then translocated into the nucleus to initiate the transcription of the targeted genes. (b) The Hedgehog pathway (left): Hh, when secreted from the other cells, binds to PTCH and allows the activation (indicated by black arrows) of SMO. SMO protein complex secretes Gli1/2 and translocates it into the nucleus, leading to the transcription of Hh-associated genes (depicted by purple arrows). (c) The Notch pathway (right): the binding of the delta ligand to the other cell; two different enzymes responsible for two different cleavages are ADAM10 or TACE and a metalloprotease that catalyzes the S2 cleavage and hence producing a substrate for S3 cleavage via the *γ*-secretase complex. Due to proteolysis, it mediates the release of NCID, which upon translocation into the nucleus starts interacting with the DNA-binding CSL protein and MAML which further activate the transcription process of the targeted genes. (d) The Wnt pathway (extreme right): Wnt ligand binds to Fz, a receptor, and induces the phosphorylation of the coreceptors, LRP5/6, which further forms the docking site for AXIN. The binding of the ligand to the receptor signals Dvl to recruit AXIN 1 along with the other kinases CK1*α* and GSK3*β* to the membrane, which interrupts the destruction complex leading to impairment of the phosphorylation of *β*-catenin and results in its destruction. Accumulated *β*-catenin then translocates from the cytoplasm to the nucleus and functions as an activator of TCF/LEF-mediated transcription of Wnt target genes.

It is a complex signaling network which consists of extracellular Hh ligands, transmembrane protein receptor (PTCH), a transmembrane protein (SMO), an intermediate transduction molecule, and the downstream molecule GLI. The role of SMO protein is to regulate the pathway positively, where PTCH plays a negative role in regulation [19]. The subtypes of GLI have different roles, where Gli1 acts in the activation of the transcription. Gli2 acts as both an activator and an inhibitor of transcription, but mainly as an activator. Gli3 inhibits transcription [95]. This signaling pathway plays an important role in the formation of the nervous system, skeleton, limbs, lungs, heart, gut, and embryonic development. In the absence of the Hh ligand, PTCH, which is present on the target cell membrane, binds to the SMO thus inhibits its activity and ultimately halts the signaling [96]. In the presence of Hh ligand, there is the spatial conformational change in the PTCH which activates the transcription factor Gli by eliminating the inhibition of SMO (Figure 5). Gli upon translocation into the nucleus regulates growth, proliferation, and differentiation of the cell [19].

Self-renewal capacity and metastasis of CSCs can also be promoted by the Hh signaling via upregulating the expression of related downstream markers of CSCs (e.g., ALDH1, Bmi-1, CCND1, CD44, C-MYC, Jagged1, Nanong, Oct4, PDGFR*α*, Snail, Twist1, and Wnt2). Directly or indirectly, some protooncogenes and suppressor genes regulate the Hh signaling in the proliferation and migration of CSCs [19]. In the case of medulloblastoma stem cells, a transcriptional repressor, BCL6, and lymphoma oncoprotein directly repress the Sonic Hh effectors Gli1 and Gli2. It involves the degradation of Gli1, due to its increased physical interaction with the *β*-catenin [96]. In the case of lung CSCs, miR-122 directly targets the Shh and Gli1 [97]. Thus, it can be suggested that the amplified Hh signaling is important for self-renewal, growth, and metastasis of CSCs.

### 5.2. JAK-STAT Signaling Pathway in CSCs

Cytokines are responsible for the stimulation of the Janus kinase/signal transducers and activators of transcription (JAK-STAT). Interleukin 2-7, granulocytes/macrophage colony-stimulating factor, growth hormone, EGF, PDGF, and interferon are the examples of some cytokines and growth factors that are responsible for transmitting the signal via this pathway. Many vital biological processes such as apoptosis, cell proliferation, differentiation, and immune regulation involve the presence of the JAK-STAT signaling pathway. The tyrosine kinase-related receptor, the tyrosine kinase JAK, and the transcription factor STAT are the three main components of this pathway. The binding site for the tyrosine kinase JAK is present in the cells. Tyrosine residues of various target proteins after binding with the ligands get phosphorylated via JAK activation, in order to achieve signaling from the extracellular to the intracellular space. The four members of JAK protein family are JAK1, JAK2, JAK3, and Tyk2, while STAT has seven members in the family (STAT1, STAT2, STAT3, STAT4, STAT5a, STAT5b, and STAT6) that play an important role in signal transduction and transcriptional activation [19].

When JAK receives the signal from the upstream receptor molecule, it activates the tyrosine kinase-related receptor and gets activated to catalyze tyrosine phosphorylation of the receptor. The phosphorylated tyrosine present on the receptor molecule acts as a signal molecule and binds to the SH2 site of STAT. Upon binding to the receptor, the tyrosine phosphorylation of STAT also occurs, resulting in the formation of a dimer that enters the nucleus and affects the expression of the targeted genes (summarized in Figure 5) and ultimately proliferates and differentiates the target cells [98].

Constitutive and abnormal activation of STAT3 and mutation in JAK2 are observed in many tumors. The self-renewal of glioma stem-like cells is promoted by HIF-1*α* via the JAK1/STAT3 pathway [99]. This pathway is activated in ALDH^high^ and CD126^+^ endometrial CSCs by IL-6, which also converts the nonstem cancer cells into cancer stem cells by activating the downstream Oct4 gene, in the case of breast CSCs [36]. A scaffold protein, AJUBA, which plays an important role in cell adhesion, differentiation, proliferation, and migration, is also responsible to promote colorectal CSC survival and proliferation through the JAK1/STAT1 pathway [100]. In the case of lung CSCs, the gene expression of JAK3 and IL-6 receptor is negatively regulated by miR-218 because microRNAs activate the JAK/STAT signaling by inhibiting the negative regulatory factor of JAK2/STAT3 [19]. Thus, some recent studies on this pathway suggest that the JAK/STAT signaling pathways play an important role in the survival, self-renewal, and metastasis of CSCs.

### 5.3. NF-*κ*B Signaling Pathway in CSCs

**Figure 6:**
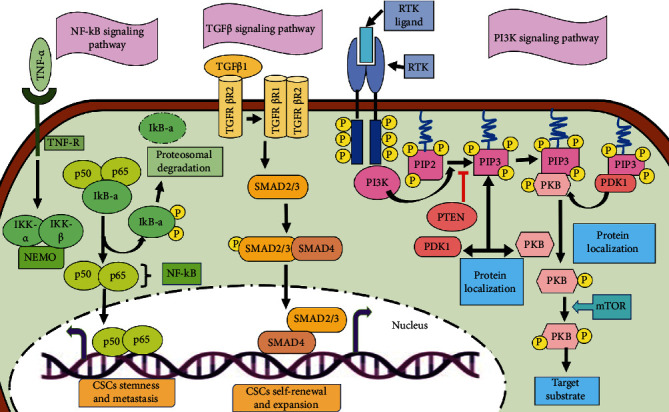
Signaling pathways involved in cancer stem cells. (a) The NF-*κ*B pathway (left): TNF-*α*, the proinflammatory cytokine, binds to the TNF receptor and induces the formation of IKK complex, which phosphorylates I*κ*B-a. Phosphorylation of I*κ*B-a results in its degradation via proteasome which leads to the accumulation of p65-p50 (acting as an NF-*κ*B) dimer into the nucleus and regulates the transcription of the targeted genes. (b) The TGF-*β* pathway (middle): TGF*β*1 ligand upon binding to the TGF-beta receptor type-2 (TGF*β*R2) promotes (indicated by black arrows) the dimerization of TGF*β*R2 with TGF*β*R1, resulting in the transphosphorylation of TGF*β*R1. The activated TGF*β*R1 further activates R-SMADs (SMAD2 and SMAD3) by phosphorylation. SMAD2/3 trimerizes with a co-SMAD (SMAD4). The SMAD trimer upon localization into the nucleus activates (indicated by purple arrows) the gene transcription and promotes cell growth and survival. (c) The PI3K pathway (right): the binding of the ligand to the RTK results in the phosphorylation of the membrane lipid PIP_2_ via intracellular PI3K and it then converts to PIP_3_. PKB bounds to its docking site in PIP_3_, and upon phosphorylation, it gets activated by various kinases involving mTOR and DNA-dependent protein kinases, which further enhances PKB-mediated phosphorylation and the activation or repression of downstream mediators. PTEN, a phosphatase that is a negative regulator (inhibition is indicated by red arrows) of this process, helps in the dephosphorylation of PIP_3_ to PIP_2_ (the black arrows are depicting pathway activation/signal propagation).

NF-*κ*B comprising five different proteins (mainly p65, RelB, c-Rel, NF-*κ*B1, and NF-*κ*B2) is the rapidly inducible transcription factor [36]. Two major pathways regulate its activity. Those pathways are the canonical NF-*κ*B and noncanonical NF-*κ*B signaling pathways. The canonical NF-*κ*B pathways activate when the ligand (e.g., cell components of bacteria, IL-1*β*, TNF-*α*, or lipopolysaccharides) binds to the receptor (such as Toll-like receptor, TNF receptor, IL-1 receptor, and antigen receptor). These receptors upon stimulation further phosphorylate and activate I*κ*B kinase (IKK protein). IKK1 proteins also get phosphorylated and activated in the noncanonical pathway by inducing the kinase (NIK) which further activates NF-*κ*B as shown in Figure 6. The IKK enzyme activity induces the production of p52 by stimulating the phosphorylation of p100 [101].

In order to activate the NF-*κ*B signaling pathway, the process of tumor development and progression produces some cytokines, proteases, and some factors responsible for growth and angiogenesis. In many cancers, overactivation of the NF-*κ*B signaling has been reported [19]. In the case of ovarian CSCs, CD44^+^ cells promote self-renewal, metastasis, and maintenance of CSCs by increasing the expression of RelA, RelB, and IKK*α* and mediate nuclear activation of p50/RelA (p50/p65) dimer [19]. The inflammatory mediator prostaglandin E2 (PGE2) activates this signaling by the EP4-PI3K and EP4-MAPK pathways which contribute to tumor formation and metastasis in colorectal CSCs [102]. miR-221/222 inhibits the expression of PTEN thereby inducing the phosphorylation of Akt which results in the increased level of p65, p-p65, and COX2 and promotes self-renewal, migration, and invasion in breast CSCs [103]. Thus, it can be determined that the elevated NF-*κ*B signaling is crucial for regulating apoptosis, proliferation, and metastasis in CSCs.

### 5.4. Notch Signaling Pathway in CSCs

The Notch signaling pathway is a highly conserved pathway involving four Notch receptors (mainly Notch1, Notch2, Notch3, and Notch4) and five structurally similar Notch ligands (Delta-like1, Delta-like3, Delta-like4, Jagged1, and Jagged2). Under some physiological conditions, binding of the delta ligand to the Notch receptor results in the expression on the neighboring cells in a juxtacrine manner. The proteolytic cleavages of the intracellular domain (ICD) of Notch by two different enzymes responsible for two different cleavages are ADAM10 or TACE (TNF-*α*-converting enzyme, also known as ADAM17) and a metalloprotease that catalyzes the S2 cleavage and hence producing a substrate for S3 cleavage via the *γ*-secretase complex (Figure 5). Due to proteolysis, it mediates the release of NCID and hence is translocated into the nucleus where it binds to the transcription factor CSL. This binding leads to the formation of transcriptional activation complex NICD/CSL that activates the targeted genes of the BHLH transcription inhibitor family [19].

Depending upon the microenvironment, Notch can act either as an enzyme, oncogene, or tumor suppressor gene. When the Notch is activated, it promotes self-renewal, metastasis, and cell survival but inhibits apoptosis. In gastric CSCs, an abundance of Delta-like ligand 4 (DLL-4) promotes tumor angiogenesis and metastasis [104]. In cervical CSCs, the Notch pathway gets activated when MAP17 (DD96, PDZKIP1), a nonglycosylated membrane-associated protein present on the Golgi apparatus and the plasma membrane, interacts with NUMB via the PDZ-binding domain. TACE/ADAM17 is activated to regulate the Notch1 signaling. They are being activated by inducible nitric oxide synthase which promotes the self-renewal capacity of CD24^+^/CD133^+^ liver cells [105]. In oral squamous cell carcinoma, the Notch1 signaling is activated to enhance the CSC-like phenotype by TNF-*α*. The migration and invasion of ovarian CSCs are induced by Notch1 when there is no sign of hypoxia [19]. Thus, these recent studies emphasize the important role played by the Notch signaling in metastasis, self-renewal, and growth of CSCs.

### 5.5. PI3K/Akt Signaling Pathway in CSCs

PI3K is an intracellular enzyme mainly phosphatidylinositol kinase that possesses a regulatory and a catalytic subunit. p85 is the regulatory subunit, and p110 is the catalytic subunit which has the activities of both the kinases serine/threonine (Ser/Thr) kinase and phosphatidylinositol kinase. Serine/threonine kinase is present as AKT which further has three isoforms (AKT1, AKT2, and AKT3), and their proteins are important effectors of PI3K because they can be directly activated after getting a response from PI3K. When the ligand binds to receptor tyrosine kinase (RTK) which phosphorylates the membrane lipid, phosphatidylinositol (3,4)-bis-phosphate (PIP_2_) via intracellular PI3K, it then converts to phosphatidylinositol (3,4,5)-tris-phosphate (PIP_3_). Protein kinase B (PKB) bounds to its docking site in PIP_3_, and upon phosphorylation, it gets activated by various kinases involving mTOR and DNA-dependent protein kinases that further enhance PKB-mediated phosphorylation and the activation or repression of downstream mediators. A conserved serine/threonine kinase, the mammalian target of rapamycin (mTOR) complex, is one of the important downstream target genes of Akt. mTOR is found to be in two different multiprotein complexes, mTORC1 (consists of mTOR, raptor, mLST8, and two negative regulators PPRAS40 and DEPTOR) and mTORC2. The role of mTORC2 is to phosphorylate Akt at serine/threonine 473 for the activation of Akt (Figure 6) [19].

The PI3K/Akt signaling is involved in cell proliferation in ovarian cancer and also in EMT [106]. Upon activation, this signaling enhances the properties of migration and invasiveness in the prostate and pancreatic cancer [107]. In the case of head and neck squamous CSCs, the activation of PI3K increases cell proliferation, migration, and invasion in ALDH^+^ and CD44^high^ cells [108]. In colorectal cancer, the expression of ALDH1 increases due to the activation of mTORC1. The expression of hepatic CSC marker EpCAM and tumorigenicity, in the hepatocellular CSCs, is upregulated upon activation of mTORC2 [36]. An inhibitor of mTOR, matcha green tea (MGT), inhibits the proliferation of breast CSCs by targeting the mitochondrial metabolism, glycolysis, and multiple signal transduction pathways [109].

### 5.6. TGF-*β* Signaling Pathway in CSCs

A TGF-*β* signaling pathway is structurally simple and regulates numerous cellular processes, such as cell proliferation, differentiation, apoptosis, and homeostasis. The TGF-*β* superfamily ligands are divided into two groups: (i) TGF-*β*/activin that consists of TGF-*β*, activin, and Nodal and (ii) BMP/GDP which includes BMP, GDF, and AMH ligands. SMAD proteins on the basis of their structure are divided into three subfamilies: R-SMADs (receptor-activated or pathway-restricted SMAD), co-SMAD, and inhibitory SMADs (I-SMADs). The ligand of TGF-*β* superfamily binds to the type II receptor and phosphorylates it. Type I receptor binds to a common pathway SMAD (co-SMAD) by phosphorylating receptor-regulated SMADs (R-SMAD). The complex of R-SMAD/co-SMAD acts as a transcription factor by accumulating into the nucleus and regulating the expression of the targeted genes [19]. The process is summarized in Figure 6.

The activated TGF/SMAD signaling is also found in human cancers. In the case of lung cancer, cellular transformation and stemness are mediated via nuclear NPM1 protein, while the TGF-*β* signaling is promoted by cancer upregulated gene 2 [110]. The role of TGF/SMAD is to proliferate CSCs; for example, in order to regulate the self-renewal of liver CSCs, the cyclin D1-SMAD2/3-SMAD4 signaling is promoted after the activation of SMAD2/3 and SMAD4 by their interaction with cyclin D1 [19]. The expression of p-SMAD2/3, SMAD4, and CD133 is induced by the upregulation of TGF-*β* in liver CSCs. In order to regulate glycolysis in glioma stem cells, the expression of PFKFB3 is upregulated by TGF-*β*1 via activating the p38 MAPK and PI3K/AKT signaling pathways [111]. SMAD7, a target gene of miR-106b, acts as an inhibitor of the TGF-*β*/SMAD signaling and inhibits the sphere formation of gastric CSCs [112]. Although there are limited studies on the TGF-*β*/SMAD signaling pathway in CSCs, this can be concluded from the earlier studies that it plays a vital role in cellular processes in CSCs.

TGF-*β* has recently been proposed as a mediator of immaturity that contributes to tumor development and relapse. It acts not only on tumor cells but also on immune system cells like dendritic cells and natural killer cells, potentiating TME's immunosuppressive property [113], whose inhibition normalises tumor stroma and makes tumors more sensitive to therapy [114], including immune checkpoint inhibitor therapy [115].

### 5.7. Wnt Signaling Pathway in CSCs

The abnormal canonical and noncanonical WNT signaling is involved in CSC survival, bulk-tumor expansion, and invasion/metastasis in a variety of human cancers [116]. On the basis of the transcriptional regulator *β*-catenin acting as a mediator, the Wnt pathway can be divided into two signaling pathways, namely, the canonical and noncanonical Wnt signaling pathways [116].

By forming the stem cell signaling network, the WNT signaling, along with other signaling cascades such as FGF, Notch, Hedgehog, and TGF/BMP, regulates the expression of functional CSC markers [116].

The canonical WNT/-catenin signaling cascade is involved in stem cell self-renewal and progenitor cell proliferation or differentiation [53, 117], whereas the noncanonical WNT signaling cascades are involved in stem cell maintenance, directional cell movement, or suppression of the canonical WNT signaling pathway [118, 119]. WNT signaling cascades, both canonical and noncanonical, play a significant role in the development and evolvement of CSCs. WNT2B, WNT3, and other canonical WNT ligands derived from cancer supporting cells or stromal cells, as well as genetic alterations in the canonical WNT/-catenin signaling components, activate the canonical WNT signaling in CSCs [120]. LGR5, which encodes an R-spondin (RSPO) receptor, is a target gene of the WNT/-catenin signaling cascade in both quiescent and cycling stem cells. Canonical WNT signals stimulate the LGR5 receptor on CSCs, allowing them to remain canonical WNT responsive and directly promote CSC proliferation by upregulating the proteins CCND1, FOXM1, MYC, and YAP/TAZ [121, 122].

WNT5A, WNT11, and other noncanonical WNT ligands secreted by cancer cells or stromal/immune cells, as well as genetic alterations that transactivate noncanonical WNT signaling cascades, activate the noncanonical WNT signaling in CSCs [116]. Through PI3K-AKT signaling activation and YAP/TAZ-mediated transcriptional activation, the noncanonical WNT signaling promotes CSC survival and therapeutic resistance [116].

In contrast, invasion and metastasis are driven by both canonical and noncanonical WNT signaling cascades. Canonical WNT/-catenin and WNT/STOP (stabilization of proteins) signaling cascades, for example, upregulate SNAI1 to repress epithelial genes, such as CDH1 (E-cadherin), for the initiation of CSC EMT, whereas noncanonical WNT signals promote CSC invasion, survival, and metastasis [116, 122, 123].

These findings indicate that the canonical WNT/-catenin signaling, as well as other WNT signaling cascades, plays a critical role in CSC malignancy.

WNT signaling cascades are the primary cause of many types of human cancers [116, 122, 123], but the development of many WNT signaling-targeted therapeutics is ground to a halt due to the complexity of WNT signaling cascades and genetic alterations in nonenzymatic signaling components. WNT signaling-targeted therapeutics in clinical trials or preclinical studies encompass anti-FZD mAb, anti-ROR1 mAb, anti-RSPO3 mAb, PORCN inhibitors, and *β*-catenin inhibitors [120].

## 6. Role of Cancer Stem Cells in Tumorigenic Processes

### 6.1. Cancer Stem Cells in Oncogenesis

The hypothesis of CSCs could be compared with the working theory for oncogenesis, that due to the accumulation of mutations in the protooncogenes or tumor suppressor genes, differentiated cells get converted into tumorigenic. These genes regulate cell growth by regulating some growth-related factors, as the overactivation of these genes can lead to uncontrolled growth and even develop cancer [124].

In order to initiate cancer, many adult stem cells, their derivative progenitor cells, or many differentiated cells can convert into CSCs. Adult stem cells are virtually present in all tissues, and due to their long-lived nature, they are prone to develop the numerous mutations than any other cells. These mutations then further lead to cancer [3]. Markers such as CD133 and ALDH1 [125] which are associated with adult stem cells are also being expressed by CSCs. It also appears that CSCs and NSCs share some similar epigenetic and genetic profiles and activated signaling pathways, such as Hedgehog, Notch, and Wnt. In the case of AML, progenitor cells can be mutated to become CSCs since they have a phenotype similar to that of progenitor cells. Due to mutation in the differentiated cells, they might acquire the properties of progenitor or stem cells and can give rise to CSCs. The first genetic or epigenetic modifications might be possible to occur in adult stem cells, and subsequent mutations may accumulate in a progenitor or more differentiated daughter cell [126]. Cell surface marker proteins found on CSCs of different tissue types such as CD133 and CD44 are most likely to be the true markers of CSCs which are involved in oncogenesis because their existence on CSCs is reproducible. However, these markers may represent some cell's ability to survive purification procedures or trigger tumor growth in mice [29].

### 6.2. Cancer Stem Cells in Tumor Growth

CSCs are suggested to participate in tumor growth, but the number of different types of CSCs involved in this process and the importance of different percentages of CSCs contained in tumors are unclear [127].

Due to the constantly evolving nature of cancer cells via genetic/epigenetic changes along with the impact of their microenvironment, any of them could become CSCs [128]. A mutation pattern found in different regions of a single tumor suggests the multiple clonal cell populations, some of which may be cancer stem cells. Moreover, various markers used to isolate CSCs can reflect the diversity of these cells [5]. Some tumor cell populations are likely to be overlooked due to the detection of CSCs, including the fact that tumor tissue samples may not be representative of the entire population.

Tumors tend to differ in the percentage of CSCs with the recorded values between 0.03% and almost 100%. This percentage is probably determined by the specific characteristics of the CSCs that initiated the tumor and by the microenvironment by controlling the frequency with which additional CSCs are produced [127]. The degree of CSC expression tends to correlate with patient prognosis since the percentage of CSCs in a tumor may reflect tumor subtype or progression level, with more CSCs generally leading to poor clinical outcome. The presence of large population of CSCs indicates the rapid proliferation rate of the tumor cells. Tumor cells are more genetically unstable as they lack the property of differentiation, and hence, it is not possible to generate differentiated progeny thus enhancing the selective advantage in context to cancer therapy [128].

### 6.3. Cancer Stem Cells in Metastasis

The term “metastasis” can be defined as the dispersion of the cancer cells via the lymphatic system or bloodstream, from the tumor it has been originated (the primary tumor) to the tissues or organs surrounding the tumor. CSCs participate in the metastasis in two ways: firstly, as the original CSCs that started primary tumor, and secondly, as CSCs derived from first one or another cell in the tumor that acquired metastatic traits. The second type of cells is more invasive than the first one and therefore more likely to metastasize due to additional genetic and epigenetic alterations.

The CSC model can be used to describe the biology of metastases and to explain the similarities between primary tumors and autologous lymph node. These similarities are in contrast to traditional cancer models which reveal that metastasis originates from monoclonal expansions of specific individual tumor subclones having specific genotypic and phenotypic features; thus, they are different from primary tumors [129]. The genetic changes that are acquired at the initial stages of tumor development are the reason for the predetermining the metastasis capacity. Because of the metastatic potential of solid tumors, the poor prognosis issue of the patients can be successfully determined using various genomic approaches and molecular signatures. Therefore, it can be suggested that the majority of cancer stem cells found in primary tumors have a metastasis gene program [130]

A subset of CSCs, which are essential for metastasis, can be found at the invasive edge of pancreatic carcinomas. CSCs are likely to aid the migration of tumor cells away from the primary tumor which is one of the prime steps in the metastatic cascade to form secondary tumors in distant organs. Thus, it can be suggested that CSCs may metastasize together along with another type of cancer cells [14]. Various genetic signatures present in CSCs might predict the recurrence and metastasis of tumor. The biomarkers such as CD133, CD44, and CD166 when combined together easily identify the risk of recurrence and metastasis in the patients suffering from colorectal cancer. The putative stem cells CD44^+^ and CD24^-/low^ can be detectable in the metastatic pleural fluid found in breast cancer and are mainly responsible for generating primary tumors in an orthotopic site and cause lung metastasis [130]. Thus, it can be concluded that CSCs undergo neoplastic growth in the new location when they metastasize [14].

### 6.4. Cancer Stem Cells in Cancer Recurrence

The role of CSCs in cancer recurrence can be found due to their tumorigenic properties and their tendency to resist many therapies like chemotherapy and radiotherapy [131]. CSCs found in breast cancer when grown in the culture are found to be resistant to the chemotherapeutic agents. Thus, after chemotherapy in breast cancer patients, there are high percentages of cells with breast CSC properties. This indicates that the treatment may have been less effective in destroying CSCs rather than removing other cancer cells. This study observed the biopsies of patients by measuring cell surface proteins. However, the fact that whether the location of these measured proteins was actually on clonally related cancer cell population, or whether there was some change in their pattern by the treatment, or whether the sensitivity of the treated cells was different in those patients who responded therapy was ambiguous. The drug-resistance mechanism of CSCs is not completely known, but it could be possible that the overexpression of antiapoptotic proteins or drug-metabolizing proteins on them could pump out the drugs out of the cell [6].

Daunorubicin and Ara-C are the examples of some chemotherapeutic drugs, to which CSCs of leukemia are resistant [132]. CSCs of various other cancers, such as pancreas or colon cancer, were also found to be resistant to chemotherapy, while some CSCs are resistant to radiation also [133, 134]. Interestingly, parthenolide and rapamycin are the drugs that kill the CSCs of AML but do not work against normal hematopoietic stem cells. A decrease in tumorigenicity was reported by temozolomide and bevacizumab when treated against CSCs in glioblastoma [135].

The use of the HER1/HER2 inhibitor lapatinib in HER2-positive breast cancer, responsible for the amplification of the HER2 gene, is the best example for eliminating CSCs by targeting a particular cancer-specific genetic alteration [131].

Most of the cases studied had revealed that CSCs could be effectively destroyed by the introduction or inhibition of specific genetic alterations. This demonstrated that an effective therapeutic approach can be achieved by the targeting of a mutation in all tumor cells and further added that in cancer recurrence, there is an involvement of both CSCs and differentiated cells [6].

## 7. Cancer Stem Cells in Evading Programmed Cell Death

Several existing therapeutics fail to eliminate tumors owing to CSCs' capacity to evade various programmed cell deaths which are all dysregulated in CSCs. As a result, establishing CSC-selective and programmed death-inducing therapeutic approaches seems to be essential.

### 7.1. Apoptosis

The proper balance of life and death signals is critical as the breakdown in control can lead to tumor growth [136]. In this aspect, apoptosis is critical in the prevention of cancer development. Many studies have demonstrated that genetic abnormalities turn normal stem cells into CSCs, permitting them to escape apoptosis and hence develop tumors [136]. Dedifferentiation and reprogramming are two alternative ways for forming apoptosis-resistant CSCs. Unfortunately, there are no therapeutics that specifically target CSCs. In addition to PI3K/Akt, NOTCH1, and Wnt/*β*-catenin, CSCs overexpress antiapoptotic proteins and possess fast DNA repair, resulting in apoptosis resilience [136] and eventually resistance to several chemotherapeutic treatments [136, 137]. Thus, while traditional anticancer medicines can reduce or eliminate cancer cells, CSCs can endure, resulting in relapses in many types of cancer or metastases by migrating beyond the initial location of the tumor [137].

#### 7.1.1. Mechanisms of CSCs to Evade Apoptosis

CSCs exhibit inherent resistance to apoptosis via a variety of mechanisms, including the overexpression of multidrug resistance transporters such as the ATP binding cassette (ABC) transporter family. ABC transporter overexpression has been documented in numerous malignancies, most noticeably in CSCs [138]. CSCs have been demonstrated to be enhanced in a variety of malignancies, leading to treatment resistance [139]. The PI3K/AKT/mTOR signaling is another route implicated in the evasion strategy of CSCs. This route is essential for cell proliferation, metabolism, invasion, survival, and thus for tumor formation and CSC maintenance [19]. Furthermore, a modulation in the ratio of apoptotic to antiapoptotic proteins is needed for the development of many cancers and contributes to the survival of CSCs. However, their role to drug resistance is not fully understood. BCL2 family proteins, which include proapoptotic proteins Bax, Bak, Bid, Bim, Bik, Noxa, and Puma as well as antiapoptotic molecules Bcl-2, Bcl-XL, and Mcl-1, were shown to be overexpressed in CSCs [140]. The altered interaction of pro- and antiapoptotic proteins is linked to CSC resistance to apoptosis and anticancer therapeutics.

Furthermore, there is a significant rise in the expression of nuclear factor erythroid 2-related factor 2 (Nrf2) in CSCs which is a redox-sensing transcription factor that supports and enhances CSC survival.

TRADD, a factor implicated in multiple receptor signaling pathways of cell survival and death, is required for NF-B activation in CSCs that in turn promotes the development of a range of inflammatory cytokines and apoptosis inhibitory protein [141]. Furthermore, lowering NF-B activation by TRADD silencing reduced cell viability, demonstrating TRADD's function in CSC survival.

XIAP, a member of the inhibitor of apoptosis (IAP) family of proteins, regulates apoptosis in CSCs and is expressed at greater levels in glioblastoma and nasopharyngeal cancer stem cells [142]. FLICE-inhibitory protein (c-FLIP), the major antiapoptotic protein responsible for resistance to chemotherapy-induced apoptosis, is overexpressed in a variety of malignancies. Its levels in CSCs, on the other hand, are substantially greater than in normal cancer cells [143], conferring resistance to TRAIL-induced apoptosis. Moreover, inhibiting cFLIP makes CSCs susceptible to TRAIL-induced apoptosis, indicating a function for cFLIP in death resistance [143].

### 7.2. Autophagy

Autophagy is required for preservation of pluripotency or the ability to self-renew and remain undifferentiated which is an essential feature of CSCs [144]. In fact, it has been discovered that CSCs from various malignancies retain a strong autophagic flux [138], and along with hypoxia, it is required for the preservation of the stem cell niche. Interestingly, Zhu et al. established that autophagy is HIF-1-dependent and essential for maintaining the balance between pancreatic CSCs and normal cancer cells [145]. As a result, autophagy is an adaptive process required for CSC maintenance.

#### 7.2.1. Mechanisms of CSCs to Evade Autophagy

Autophagy and autophagy proteins are elevated in breast CSCs [146]. Its failure has a deleterious impact on the expression of staminal markers and, hence, the ability to self-renew in a variety of CSCs.

The basic pathways of autophagy-dependent CSC maintenance have been [147] demonstrated to occur via the EGFR/Stat3 and TGF/Smad pathways in breast cancer stem-like cells. Inhibiting autophagy in triple-negative breast CSCs inhibited the STAT3/JAK2 pathway-mediated production of IL-6, a cytokine critical for CSC survival and required to produce the CD44^+^/CD24^low^ phenotype in breast cancer cell lines [148]. As a result, the IL-6-JAK2-STAT3 pathway appears to play an important role in the conversion of non-CSCs to CSCs.

FOXO proteins that govern autophagy affect cancer development and metastasis as well as the fate of CSCs [149], which needs to be explored. FOXO3 inhibition resulted in long-term CSC self-renewal in many malignancies [150]. However, further research is needed to understand how FOXO-dependent control of stemness and autophagy pathways are linked in carcinogenesis. The study on ovarian CSCs showed a link between autophagy and stemness [151]. Forkhead Box A2 (FOXA2) has been reported to be overexpressed in ovarian CSCs and is regulated by autophagy. Moreover, inhibition of autophagy by pharmacological and genetic techniques results in FOXA2 decrease and, as a result, loss of self-renewal potential. Several studies have shown that autophagy plays a function in chromosomal stability management; hence, CSCs may activate autophagy to avoid additional DNA damage and thus maintain their survival [152].

## 8. Therapeutic Strategies to Target CSCs

CSCs have been found to influence the tumor metastasis and drug resistance. By targeting CSCs, the problem of the poor prognosis of the patients can be overcome leading to increased survival rates of the patient. There are several ways or approaches to target CSCs; some of them are explained in the following section.

### 8.1. Targeting CSC Niche

The main properties of the CSC microenvironment are inflammatory cytokines, hypoxia, and the regulation of the potency of self-renewal, proliferation, and differentiation via the perivascular niche. Several inflammatory cytokines (e.g., IL-1*β*, IL-6, and IL-8) are responsible for the activation of various pathways such as STAT3 and NF-*κ*B in tumors as well as stromal cells. The process of angiogenesis, metastasis, and self-renewal is promoted by the secretion of cytokines via the aforementioned pathway in a positive feedback loop. It has been observed that blocking of the IL-6/IL-8 cytokine signaling leads to decrease in tumor growth [153]. Repertaxin which is known to be a noncompetitive inhibitor of the IL-8 and CXCR1 signaling is responsible for reducing tumor size and increasing the efficacy of chemotherapy. Plerixafor (AMD3100), a drug that targets CXCR4, is an effective mobilizer for HSC and used in the treatment of the patients suffering from multiple myeloma and non-Hodgkin lymphoma (NHL) [154].

Hypoxic microenvironment results in the activation of HIFs which resist cellular differentiation and chemotherapy/radiotherapy. Moreover, it also modulates angiogenesis and apoptosis [155]. Few small molecules acting as inhibitors of the HIF pathway approved by the Food and Drug Administration (FDA) are bortezomib (Velcade®, PS-341) approved in 2003 against multiple myeloma and temsirolimus (Torisel®, CCI-779) approved in 2007 against renal cell carcinoma. Bevacizumab (Avastin®), cediranib (AZD2171), sunitinib, and vandetanib are examples of some other drugs which inhibit angiogenesis by blocking the vascular endothelial growth factor (VGEF) that accounts for the migration of the endothelial cells. These drugs also suppress the self-renewal capacity of CSCs and thus inhibiting tumor propagation and metastasis [156].

### 8.2. Targeting CSC Signaling Pathway for Cancer Therapy

The targeting CSC signaling pathways involved in self-renewal, proliferation, and differentiation, in order to maintain the stem cell properties, offers new avenues for cancer treatments. Therefore, targeting the essential pathways, such as Notch, Wnt, and Hedgehog (Hh), can block the self-renewal potency of CSCs [19]. Disulfiram, an antialcoholism drug in the case of breast CSCs, inhibits TGF-*β*-induced metastasis by the ERK/NF-*κ*B/Snail pathway. Vismodegib, approved by European Medicines Agency (EMA) in 2013 and US FDA in 2012, is a drug that targets the Hh pathway and is used against the therapy of those metastatic basal cell carcinoma (BCC) patients whose surgery and radiotherapy could not be done [157]. BMS-833923, saridegib (IPI-926), sonidegib/erismodegib (LDE225), PF-04449913, LY2940680, LEQ 506, and TAK-441 were used as monotherapy. Vantictumab (OMP-18R5) is a mAb that blocks the Fz receptors (such as Fz1, Fz2, Fz5, Fz7, and Fz8) [57] and reduces proliferation of tumor cell and tumor-initiating cell number in tumors of the lung, breast, colon, and pancreas.

Identification of molecular mechanisms and signaling pathways characteristics for CSCs in solid and hematologic malignancies has been the focus of numerous studies. Notch, Hedgehog, Wnt, and NF-*κ*B cascades have been reported to be dysregulated in cancers and are linked with high proliferative multidrug resistance. These associations serve as potential targets for CSC specific eradications. However, further studies are required to determine the safety of these targeted therapies because these signaling pathways are also crucial for normal stem cell maintenance [19].

### 8.3. Targeting CSCs for Immunotherapy

Various experiments have been conducted, focusing on how the immune system plays a role in preventing tumor growth. However, cancer cells' escape mechanisms from the immune system have also been studied. Along with chemotherapy and radiotherapy, CSCs are found to be resistant to immune therapy also. The evidence can be found in many cases: the absence of the expression of major histocompatibility complex class I leads to easy escape from T lymphocytes [158].

Cancer immunotherapy mainly targets the growth and expansion of cancer cells by recognizing them via the immune system. Some cell surface molecules which are being expressed on CSCs play a major role in identifying particular antigen and detecting specific targets for CSC immunotherapies [19], e.g., ALDH, CD44, CD133, EpCAM, and HER2. Immune checkpoints act as the endogenous regulators of the immune response. They also play a role to limit the autoimmunity as they mediate coinhibitory signaling pathways. An example of such immunoinhibitory pathways is CTL antigen 4 (CTLA-4/B7) or the programmed cell death-1 (PD1/PDL1). These are the negative immune regulatory pathways that have been identified as protecting cancer cells from being killed by immune cells [153]. Recently, several novel anti-CSC immunotherapy strategies have been developed, such as immunological checkpoint blocking or chimeric antigen receptor-T (CAR-T) therapies [159]. CAR-T cells are engineered T cells possessing an artificial receptor specific for tumor-associated antigens (TAAs) through which they perfectly target and eradicate cancer cells.

In preclinical studies, CSC markers used in CAR-T cell therapies include CD20, CD44, c-Met CD133, CD166, CD38, CLL-1, CD123, EpCAM, CD171, ROR1, CD47, and CD117. Moreover, many of them have recently undergone clinical trials, resulting in substantial cancer regression [159].

Some drugs are approved by the FDA that target checkpoint receptors of the immune system and have shown effectiveness in the cancer patients, e.g., nivolumab, pembrolizumab, cemiplimab (CTLA-4, PD-1), avelumab, durvalumab, and atezolizumab (PD-L1) [19].

In order to enhance the efficacy for treating cancer, many effectors capable of recognizing and killing CSCs are also targeted for immunotherapy, such as cells involved in innate immunity (e.g., natural killer (NK) cells and *γδ*T cells), antibodies involved in acquired humoral immunity, and cells in acquired cellular immunity (e.g., CSC-based dendritic cells and CSC-primed cytotoxic T lymphocytes) [137].

### 8.4. Metformin: An Antidiabetic Drug Targeting CSCs

Metformin (*N*′, *N*′ − dimethylbiguanide), a well-known antidiabetic drug used for treating the patients of type 2 diabetes mellitus (DM), is an oral hypoglycemic agent. It acts by downregulating hepatic gluconeogenesis thus reduces blood glucose level and upregulates the uptake of glucose in the peripheral tissues [160].

It has been observed that metformin possesses antitumor effects as its consumption reduces the chances of breast and pancreatic cancers in the patients suffering from DM, but the mechanism of drug action is still unknown. The increase in insulin sensitivity caused by metformin inhibits cancer cell growth by activating AMP kinase (AMPK), which then inhibits the PI3K/Akt/mTOR signaling pathway through mTOR phosphorylation resulting in rapid inhibition of protein synthesis and cell growth [161]. By regulating various processes, such as expression of cyclin D1-mediated cell cycle, p53, and phosphorylation in breast cancer and pancreatic cancers, metformin can directly suppress the tumor growth and cell proliferation. By inactivating the NF-*κ*B pathway and HIF-1*α*, metformin can reduce the production of inflammatory cytokines (e.g., IL-6, TNF-*α*, and VEGF). Metformin's antitumor activity in vitro and in vivo may also be associated with inhibition of the insulin/IGF-1 pathway via AMPK activation by inactivation of breast CD44^+^/CD24 CSCs and the EMT phenotype, or even with inhibiting cellular proliferation, clonogenic potential, migration/invasion, and CSC self-renewal capacity in gemcitabine-resistant pancreatic cancer cells [162]. Metformin also functions as an immunomodulator by activating AMPK and blocking the HIF-1 pathway, which reduces CD39/CD73 expression-dependent MDSC immunosuppressive activity in ovarian cancer patients and boosts antitumor T cell immunological responses [163].

Metformin can considerably reduce microvascular density (MVD), improve vascular normalization, and suppress tumor angiogenesis in metastatic breast cancer models, with downregulation of platelet-derived growth factor B (PDGF-B) playing a key role in this process [164]. Moreover, restoration of normalization in the tumor vasculature further increases the sensitivity of CSCs into therapy [165]. Recently, Seo et al. [166] evaluated the effect of the mevalonate (MVA) pathway on the metformin-induced tumor suppression as this pathway results in the synthesis of sterols and protein prenylation, both of which play a pivotal role in tumor growth. They found that in the case of colorectal cancer (CRC), metformin acts as a negative regulator of the mevalonate pathway and shows inhibitory effects on CSCs. The increased expression of the MVA pathway enzymes (e.g., FDPS, GGPS1, HMGCR, and SQLE) was reversed upon adding mevalonate. Metformin also suppressed CSCs by inhibiting the processes of protein prenylation via geranylgeranylation and farnesylation that occurs via the MVA pathway.

The expressions of CSC surface marker CD44 and EpCAM; CSC genes such as EZH2, Notch1, Nanong, and Oct4; and miRNA of let-7 and miRNA-200 family in the CSC-like sphere cells of gemcitabine-resistant cells have also been found to be inhibited by metformin [167]. Overall, these data can suggest that metformin possesses antitumor effects and may commit to targeting CSCs.

### 8.5. Targeting CSCs via Nanotechnology

It is reported that the CSCs promote tumor growth and are highly resistant to traditional therapies, such as chemotherapy and radiotherapy, leading to tumor relapse and metastasis. Therefore, the use of nanotechnology along with tumor biology is of great importance, such that nanosized materials may be used for anticancer therapies driven by CSC [168].

Nanotechnology is a branch of science concerned with the study of devices with dimensions ranging from 1 to 1000 nanometers. Recently, several nanostructures from organic as well as inorganic materials have been used in either passive or active targeting of tumors for cancer therapy and diagnosis. A novel conception of nanovesicles, polymeric micelles, liposomes, dendrimers, and polymeric nanoparticles (NPs) can access a solid tumor tissue via the porous structure of a tumor vascular system and selectively deliver therapeutic agents to the targeted sites [8]. The surface of the nanoparticles has been developed to target CSCs accurately and effectively. Due to their magnetic property, the nanoparticle may be concentrated in the tumor region and the particular drug or monoclonal antibody can be targeted and released directly at the tumor site without affecting any other parts of the body [8].

Numerous nanoparticles with different sizes can be easily prepared by modifying their surfaces to target CSCs, such as allotropes of carbon (e.g., nanodiamond, graphene, and carbon nanotubes), noble metal (e.g., gold nanoparticles), organic polymers, and liposome nanoparticles. Graphene oxide (GO) is a graphene derivative with carbon atoms linked to oxygen functional groups, giving it exceptional chemical versatility. As a result, graphene's surface can be easily modified with various biochemical molecules and agents of interest, making graphene an excellent carrier of drugs or nucleic acids for the targeted cancer therapies. GO specifically targets a global phenotypic property of CSCs, and it has the potential to reduce the number of genuine CSCs by inducing differentiation and inhibiting proliferation. Carbon nanotubes on the other hand are the cylindrical nanostructure of graphene that are mainly used in carbon nanotube-mediated thermal treatments for the elimination of both the differentiated cells that are responsible for creating the bulk of tumor and the breast cancer stem cells that promote growth and recurrence of tumors. Nanodiamonds are the truncated semioctahedral carbon structure that are found to be the very efficient nanomedicine-based approach for overcoming chemoresistance in hepatic CSCs after forming a nanodiamond drug complex by the process of physical adsorption of epirubicin on nanodiamonds [169]. Gold nanoparticles are used as nanovectors for translational purposes, as they are biocompatible as well as nontoxic. To target glioblastoma CSCs, gold NPs are coupled with a peptide recognizing CD133. PEGylated gold NP functions efficiently with an anti-CD44 antibody to target breast or gastric CSCs. To target CSCs, organic nanoparticles such as liposomes and polymeric nanoparticles are also used [169]. Disulfiram, an antialcoholism drug as well as an NF-*κ*B inhibitor, when combined with copper *in vivo*, is employed in a liposome to target CSCs and reversing the process of chemoresistance [170].

Recently, nanotech-based drugs have been the interest of many researchers because of their effectiveness in developing anticancer therapies and targeting CSCs. The examples of some clinically approved nanomedicines are albumin-bound paclitaxel particles (Abraxane), iron oxide nanoparticles (nanotherm), methoxy-PEG-poly(d,l-lactide)-paclitaxel micelle (gene-xol-PM), PEG-1 asparaginase (Oncaspar), PEGylated liposome (Doxil), and SMANCS (zinostatin) [171]. The nanotech-based anticancer drugs could help in the treatment and prevention of various types of cancers because of their excellent diffusion capacity and effectiveness against various tumors and CSCs. Furthermore, nanomedicines used to target CSCs have several advantages, which include enhanced cell absorption, greater systemic circulation, improved biodistribution profiles, and the ability to address the problems of low stability and solubility with minimal side effects [8].

## 9. Conclusions

A subset of a distinct group of CSCs is related to the tumor phenotype characterized by the enhanced cell survival rate, invasive and metastatic ability, resistance to treatment, and recurrence of tumors which can lead to poor prognosis. As the environmental factors affecting CSC niche are not well understood, therefore, there is still a lot of scope for improving the current methods used for isolating, identifying, and targeting CSCs. Numerous novel anti-CSC immunotherapy strategies, such as chimeric antigen receptor-T cell therapeutics, are increasingly being optimized for improving the specificity and clinical outcomes leading to the reduction of adverse side effects in cancer patients [159]. Targeting tumor cells via chemotherapy results in the emergence of drug-resistant tumor cells which probably originate from CSCs. This is due to the CSC model, which states that CSCs divide symmetrically to replenish the CSC pool and asymmetrically to generate daughter cells (non-CSCs) with low tumorigenic potential. However, due to transcriptional, epigenetic, or environmental changes, non-CSCs can undergo a dedifferentiation process to acquire stem-like characteristics and get reprogrammed towards a more aggressive tumorigenic fate. Thus, the dedifferentiation of non-CSCs into CSCs can make latter resistant to various conventional therapies. To eliminate the chances of tumor recurrence, there is a need for precise diagnostic and screening methods that could detect and monitor the residual CSCs who have escaped the conventional therapy. The novel approach of combining small molecules and immunotherapies with traditional chemotherapeutic drugs that specifically target CSCs only could provide a better direction for the treatment of cancer patients.

As discussed in this review, both NSCs and CSCs contain a variety of biomarkers and signaling pathways. Therefore, all the regulatory factors cannot be used as therapeutic targets that contribute to CSCs. Effective targeting of CSCs without destroying the normal cells needs some of the novel approaches for identifying the realistic drug targets specific to CSCs only. Besides, factors responsible for the stemness of CSCs should also be considered. Still numerous challenges must be overcome to effectively target CSCs, such as exploration of tumor-specific characteristics of CSCs, lack of models that recapitulate the biological complexity of tumors, and obstacles in mimicking the CSC-specific niche. Moreover, most interesting question in the therapeutic use of cancer is whether CSCs should be triggered or hindered. As a matter of fact, various promising strategies for suppressing tumor relapse and metastasis in terms of targeting specific CSCs of a specific cancer must be investigated in order to achieve successful CSC-targeted therapies and thus improve cancer patient survival rates.

## Figures and Tables

**Table 1:** Similarities between NSCs and CSCs [5, 7, 9, 10].

Common features associated with NSCs and CSCs
(i) Unlimited proliferation potential and self-renewal property.

(ii) Regulation of their self-renewability by using common signaling pathways like Wnt/*β*-catenin, Notch, and Shh.

(iii) Expression of similar surface receptors such as CD133, CXCR4, and a6 integrin.

(iv) Share same telomere-lengthening mechanisms for replicative ability.

(v) Possess high nuclear to cytoplasmic ratio as well as increased expression of antiapoptotic genes.

**Table 2:** Theories suggesting origin of CSCs [11, 12].

Theories	Explanations
CSCs derived from normal stem cells	This theory suggests that for promoting their self-renewal, cancer cells utilize the regulatory pathways of existing stem cells.
	In comparison to mature differentiated cells, the characteristic property of self-renewal provides a longer life span to stem cells.
	Therefore, hypothetically mature cells with limited life span do not undergo multiple mutations which are essential for tumor formation and metastasis.

CSCs derived from progenitor cells	Progenitor cells having the partial ability for self-renewal are more abundant in the adult tissue than the stem cells which form the basis of this hypothesis.

CSCs derived from differentiated cells	It suggests that there is the probability that a tissue which has enough population of differentiated cells could undergo an essential sequence of events for dedifferentiation. These differentiated cells upon induction of epithelial-mesenchymal transition (EMT) acquire stem cell-like phenotype and formation of CSCs.

**Table 3:** Dissimilarities between NSCs and CSCs [5, 7, 9, 10].

Characteristic features	Normal stem cells	Cancer stem cells
Occurrence	Present in the small percentage among all adult normal tissues and organs like skin/hair follicles, heart, and mammary glands	Present in small percentage within the tumor in the human breast, lung, liver, gall bladder, and brain cancers
Origin	Derived from embryonic and adult stem cells	Not specific. They are likely to have arisen from the pool of normal stem or their precursor in tissues following mutations
Self-renewal property	Extensive proliferative potential with limited growth	Extensive proliferative potential with indefinite growth
Differentiation property	Highly regulated	Highly dysregulated that can initiate tumorigenesis
Stem cell niche	Supportive and provides homeostasis maintenance	Altered and deregulated due to dominant proliferation-promoting signals
Chromosomal arrangement	Stable and normally diploid with relatively long telomeres	Aneuploid having short telomeres
Therapeutic treatments	Moderately sensitive	Highly resistant
Function	Maintaining tissue homeostasis and regenerating damaged tissue	Recurrence and progression of tumor Providing resistance against cell death and conventional therapies

**Table 4:** Markers used for identification of CSCs in tumors [19].

Cancer type	Markers for CSCs	Molecular weight (kDa)	Chromosomal location (in human)	Description of the markers	References
Acute myeloid leukemia	CD10^+^	100	3q25.2	Inhibits peptide hormones like glucagon, bradykinin, and oxytocin.	[31]
	CD19^+^	95	16p11.2	Involved in class of molecules of signal transduction and regulates differentiation of B-lymphocyte.	[32]
	CD20^+^	33-37	11q12.2	Helps in the development of plasma cells from the differentiated B cells.	[33]
	CD34^+^	105-120	1q32.2	Helps in the attachment of stem cells to bone marrow.	[34]
	CD38^−^	42	4p15.32	Acts as an intracellular messenger for Ca^2+^ mobilization and also acts as a prognostic marker for patients suffering from chronic lymphocytic leukemia.	[19]
	CD44^+^	85-250	11p13	Cell surface protein when targeted leads to the eradication of leukemic stem cells.	[35]
	CD45RA^+^	205-220	1q31.3-q32.1	Acts as a class of activation regulators for leukocytes.	[36]
	CD71^+^	190	3q29	Acts as a transferrin receptor essential for the development of nerve.	[37]
	CD123^+^	70	Xp22.3 and Yp13.3	Acts as an interleukin-specific subunit of the cytokine receptor which is heterodimeric.	[38]

Brain cancer	A2B5^+^	—	—	Acts as a monoclonal antibody specific of polysialogangliosides and to a lesser extent of polysialoproteins. It helps to identify the subpopulations of nerve cells in the CNS.	[19]
	CD36^+^	85	7q21.11	Acts as the main glycoprotein, is present on the surface of platelet, and functions as an adhesion molecule.	[39]
	CD44^+^	85-220	11p13	Acts as a glycoprotein, which gets knockdown in glioblastoma xenograft models resulting in the inhibition of cell growth and improved response to chemotherapy.	[40]
	CD49f^+^	125	2q31.1	Acts as a subunit of the family of laminin receptors, which has been used to detect TICs.	[41]
	CD90^+^	25-35	11q23.3	Acts as a glycoprotein that is required for T cell adhesion and signal transduction.	[19]
	CD133^+^	115-125	4p15.32	Acts as a transmembrane glycoprotein that forms spheres, produces tumors *in vivo*, and exhibits the property of chemoresistance.	[42]
	EGFR^+^	170-180	7p11.2	Promotes proliferative migration in the tumors by binding to the epidermal growth factor.	[43]
	L1CAM^+^	200-220	Xq28	Acts as an adhesion molecule and is important for development, neuronal migration, and differentiation of the nervous system.	[19]

Breast cancer	ALDH^+^	54	9q21.13	Acts as an enzyme responsible for providing resistance to cell.	[44]
	CD44^+^/CD24^−^	85-250/35-45	11p13/6q21	Glycoprotein plays a role in the process of cellular migration and self-renewal. During metastasis, it increases blood flow in the tumor.	[45]
	CD49f^+^	125	2q31.1	Acts as a protein of the integrin family, which is present on the membrane and is responsible for signaling and cell surface adhesion.	[19]
	CD90^+^	25-35	11q23.3	Acts as a glycoprotein that is required for T cell adhesion and signal transduction.	[19]
	CD133^+^	115-125	4p15.32	Lipid composition in the cell membranes is maintained by this transmembrane glycoprotein.	[46]
	CD44^+^	85-250	11p13	Monitors variations in ECM and therefore influences cell growth, survival, and differentiation.	[47]
	CD44v6^+^	85-250	11p13	Is responsible for cellular migration and adhesion.	[48]

Cervical cancer	ABCG2^+^	72	4q22.1	Is among the largest families of transmembrane proteins that is implicated in providing resistance to camptothecin analogues and mitoxantrone.	[49]
	ALDH^+^	54	9q21.13	Marker possesses the ability to self-renew and differentiate and has enhanced tumorigenicity.	[46]
	CD49f^+^	125	2q31.1	In the presence of this marker, the cells can self-renew, enhance tumorigenic capabilities, and increase resistance to ionizing radiation.	[49]
	CD133^+^	115-125	4p15.32	Acts as a glycoprotein having 5 transmembrane domains that help in detecting the tumor.	[49]
	CD44^+^	85-250	11p13	Acts as a marker that causes tumor invasiveness and metastasis.	[50]
	CD49f^+^	125	2q31.1	Acts as a subunit of the family of laminin receptors, which has been used to detect TICs.	[41]
	CD133^+^	115-125	4p15.32	Acts as a biomarker that plays a role in cell-cell and cell-matrix contact formation.	[51]
	CD166^+^	100-105	3q13.11	Binds to CD6 which is a T cell differentiation antigen and plays a role in cell adhesion and migration.	[19]
	CD200^+^	45-50	3q13.2	Acts as a glycoprotein which is regulating immunosuppression and antitumor activity.	[19]
	CD206^+^	162-175	10p12.33	Acts as a mannose receptor that plays a major role in endocytosis, phagocytosis, and immune homeostasis	[52]
	EpCAM^+^	40	2p21	Acts as an homotypic cell adhesion molecule which is calcium-independent and can be expressed on normal epithelial cells and gastrointestinal cancers.	[53]

Cutaneous squamous-cell carcinoma (cSCC)	CD44^+^	85-250	11p13	Acts as a putative tumor cell surface marker with increased concentration in subpopulation of SCC.	[54]
	CD133^+^	115-125	4p15.32	Transmembrane glycoprotein and its upregulation play an important role in tumorigenic processes and development of CSCs.	[55]

Esophageal cancer	ALDH^+^	54	9q21.13	Acts as an intracellular enzyme that helps in detoxifying aldehydes and regulating the conversion of retinoic acid from retinol.	[56]
	CD44^+^	85-250	11p13	Receptor that acts as an activator for the tyrosine kinase receptor, thus increases tumor cell proliferation via MAPK.	[56]
	CD90^+^	25-35	11q23.3	Acts as a surface glycoprotein whose expression can lead to tumor heterogeneity and malignancy.	[19]
	CD133^+^	115-125	4p15.32	Acts as a transmembrane glycoprotein that forms spheres, gives rise to tumors *in vivo*, and exhibits chemoresistance properties.	[42]

Gall bladder cancer	CD44^+^/CD133^+^	85-250/115-125	11p13/4p15.32	Act as the potential markers of CSCs and detect their expression in primary GBC as well as in the GBC-SD cell line.	[57]

Head and neck squamous cell carcinoma	ALDH^+^	54	9q21.13	Acts as an intracellular enzyme that refines for cancer stem cells and involved in EMT.	[13]
	CD44^+^	85-250	11p13	Acts as a cell-surface glycoprotein, acts as a receptor for hyaluronic acid, and is involved in the process of cell adhesion and migration associated with tumor progression and metastatic spread of HNSCC.	[13]
	CD133^+^	115-125	4p15.32	Transmembrane glycoprotein and cells possessing this glycoprotein found to have high clonogenicity, invasiveness, and tumorigenicity and are also resistant to paclitaxel.	[13]

Laryngeal cancer	ALDH^+^	54	9q21.13	Intracellular enzyme and cells using it as a biomarker possess increased potential to proliferate.	[58]
	CD44^+^	85-250	11p13	Acts as a cell surface glycoprotein whose overexpression signifies the aggressiveness and a prognostic factor in LC.	[58]
	CD133^+^	115-125	4p15.32	Acts as a putative CSC marker and is also identified in the human laryngeal tumor Hep-2 cell line as a marker of CSCs.	[58]

Liver cancer	CD13^+^	150-170	15q26.1	Acts as a receptor of human coronavirus strain, causing infection in upper respiratory tract and leukemia.	[59]
	CD24^+^	35-45	6q21	Acts as a marker that during metastasis, it increases blood flow in the tumor.	[60]
	CD44^+^	85-250	11p13	Acts as a glycoprotein that plays a role in the process of cellular migration and self-renewal.	[61]
	CD133^+^	115-125	4p15.32	Acts as a transmembrane glycoprotein that forms spheres, produces tumors *in vivo*, and exhibits chemoresistance properties.	[42]
	CD206^+^	162-175	10p12.33	Acts as a biomarker that can predict the progression of liver cancer.	[52]
	EpCAM^+^	40	2p21	Regulates EMT, stemness, and metastasis of cells via the PTEN/AKT/mTOR pathway.	[62]
	OV-6^+^	—	—	Acts as a mouse monoclonal antibody raised against isolated hepatic oval cells. It acts as a marker for oval cells in rat and hepatic stem cells.	[19]
	CD44^+^	85-250	11p13	Acts as a transmembrane glycoprotein that is involved in various processes like invasion, migration, and adhesion.	[63]
	CD87^+^	32-56	19q13	Acts as a receptor used to activate urokinase plasminogen and affecting many normal and pathological processes which are associated with plasminogen activation of the cell surface and local degradation of extracellular matrices.	[19]
	CD90^+^	25-35	11q23.3	Surface molecular marker of CSCs and cells possessing this marker have higher proliferation, self-renewal, and tumorigenic capacity.	[64]
	CD133^+^	115-125	4p15.32	Acts as a transmembrane glycoprotein that forms spheres, produces tumors *in vivo*, and exhibits chemoresistance properties.	[41]
	CD166^+^	100-105	3q13.11	Expressed on the cell surface by interacting with the tumor cells via heterotypically or homotypically.	[65]

Malignant mesothelioma	CD9^+^	24	12p13.31	Acts as a glycoprotein responsible for differentiation, adhesion, and signal transduction in normal cell and movement and metastasis in cancer cell.	[66]
	CD24^+^	35-45	6q21	Acts as a marker which is present on the cells and leads to proliferation via an asymmetric cell division-like manner.	[67]
	CD26^+^	110	2q24.2	Acts as an intrinsic membrane-bound glycoprotein and a member of serine exopeptidase family.	[67]

Melanoma	ALDH^+^	54	9q21.13	Intracellular enzyme and those cells using it as a biomarker possess increased potential to proliferate.	[68]
	CD133^+^	115-125	4p15.32	Acts as a transmembrane glycoprotein that forms spheres and generates tumors *in vivo* and possesses chemoresistance properties.	[69]
	CD271^+^	45	17q21.33	Acts as a receptor for nerve growth factor and mediates cell proliferation and nerve cell death.	[70]
	CD27^+^	50-55	12p13.31	Acts as a transmembrane glycoprotein that controls B cell activation and the production of immunoglobulins.	[71]
	CD138^−^	92	2p24.1	Is a part of the syndecan proteoglycan family that involves in cell proliferation and differentiation and association between cells and matrices.	[72]

Nasopharyngeal cancer	ALDH^+^	54	9q21.13	Intracellular enzyme and those cells using it as a biomarker possess increased potential to proliferate.	[73]
	EpCAM^+^	40	2p21	Regulates EMT, stemness, and metastasis of cells via the PTEN/AKT/mTOR pathway.	[62]
	CD44^+^	85-250	11p13	Monitors variations in ECM and therefore influences cell growth, survival, and differentiation.	[74]
	CD133^+^	115-125	4p15.32	Glycoprotein is expressed in many tumor cells lines.	[75]

Oral squamous cell carcinoma (OSCC)	CD44^+^/CD24^−^	85-250/35-45	11p13/6q21	Receptor used for hyaluronic acid and it acts as an activator for the tyrosine kinase receptor, thus increases proliferation of tumor cells via MAPK.	[76]
	ITGA7^+^	128.9	12q13.2	Acts as an integrin responsible for metastasis, cell migration, morphogenesis, and differentiation, and during the process of myogenesis, it plays a role in differentiation and migration.	[77]

Ovarian cancer	ALDH^+^	54	9q21.13	Only a detectable marker was expressed in all primary tumors.	[78]
	CD24^+^	35-45	6q21	Acts as a biomarker which defines an ovarian cancer-initiating cell population.	[79]
	CD44^+^/CD117^+^	85-250/	11p13	Acts as a class of transmembrane receptors classified as stem cell factors.	[19]
	CD133^+^	115-125	4p15.32	Acts as a transmembrane glycoprotein that forms spheres and generates tumors *in vivo* and possesses chemoresistant properties.	[19]

Pancreatic cancer	ABCG2^+^	72	4q22.1	Acts as a membrane protein which is a part of ABC transporters and involves in the drug-resistant properties of CSCs.	[80]
	ALDH^+^	54	9q21.13	Is associated with the tumorigenic cells present in the pancreatic ductal adenocarcinoma.	[80]
	CD44^+^/CD24^+^/EpCAM^+^	85-250/35-45/40	11p13/6q21/2p21	Acts as a biomarker present on the cells which possesses the ability to form tumors.	[19]
	CD133^+^	115-125	4p15.32	Acts as a transmembrane glycoprotein that forms spheres and generates tumors *in vivo* and possesses chemoresistant properties.	[42]
	CXCR4^+^	40	2q22.1	Is associated with the patient's prognosis having pancreatic tumors and can be used for targeting tumors.	[81]

Prostate cancer	*α*2*β*1^+^	160	5q11.2	Acts as a receptor required for cell adhesion and recognition.	[82]
	ALDH^+^	54	9q21.13	Acts as an enzyme whose increased activity can be used for isolating human prostate cancer cells with enhanced properties of clonogenesis and migration *in vitro* as well as increased tumor- and metastasis-initiating capacity *in vivo*.	[83]
	CD44^+^	85-250	11p13	Acts as a receptor used for hyaluronic acid, and it acts as an activator for the tyrosine kinase receptor, thus increases proliferation of tumor cells via MAPK.	[84]
	CD166^+^	100-105	3q13.11	Acts as a surface marker used for enrichment of both murine and human prostate tissue stem or progenitor cells on the basis of *in vitro* sphere formation and *in vivo* tissue regeneration.	[85]
	CD133^+^	115-125	4p15.32	Acts as a cell surface marker used to identify CSCs in prostate cancer cell lines.	[86]
	CXCR4^+^	40	2q22.1	Acts as a receptor for CXC chemokine function with CD4 protein to facilitate the entry of HIV into cells.	[19]
	E-cadherin^+^	75-80	16q22.1	Acts as a key permissive factor that enables *in vitro* invasion of cancer stem cell.	[87]
	EZH2^+^	43.5	7q36.1	Acts as a member of polycomb family which is essential in the CNS.	[19]

Renal cell carcinoma (RCC)	ALDH^+^	54	9q21.13	Acts as a biomarker that promotes formation of sphere, clonogenicity, proliferation, and invasion of the cells.	[42]
	CD44^+^	85-250	11p13	Promotes various signaling pathways including activation of MAPK, PI3K/AKT, RTK, and TGF*β*, via supporting cellular proliferation, survival, and invasion.	[42]
	CD105^+^	90	9q34.11	Acts as a receptor present in the TGF-*β* signaling and is responsible for angiogenesis.	[19]
	CD133^+^	115-125	4p15.32	Acts as a transmembrane glycoprotein that produces spheres by giving rise to tumors *in vivo* and possessing chemo-resistant properties.	[42]
	CXCR4^+^	40	2q22.1	Acts as a G protein-coupled receptor (GPCR) with the seven transmembranes on the cell membrane known to be part of cell-stroma interactions leading to a permissive niche for metastasis.	[42]

Stomach cancer	ALDH^+^	54	12q24.12	Acts as a marker that generates chemoresistance via the Notch1 and Shh signaling.	[88]
	CD24^+^	35-45	6q21	Acts as a cell surface protein that acts as a ligand for P-selectin, which is expressed on the cell surfaces of activated platelets and endothelial cells, in the process of tumor dissemination.	[89]
	CD44^+^	85-250	11p13	Cell surface receptor plays a crucial role in degradation of matrix, proliferation, and cell survival.	[19]
	CD44v8-10^+^	85-250	11p13	Is derived from CD44 having a specific class of CSCs.	[19]
	CD49f^+^	125	2q31.1	Acts as a subunit of the family of laminin receptors, which has been used for identifying TICs.	[41]
	CD54^+^	75-115	19p13.2	Acts as an adhesion molecule expressing in tumor cells which are malignant.	[19]
	CD71^+^	190	3q29	Acts as a transferrin receptor acting as a carrier protein that transports iron within the cell and helps in maintaining the cellular iron homeostasis.	[90]
	CD90^+^	25-35	11q23.3	Acts as a membrane GPI-anchored protein.	[91]
	CD133^+^	155-125	4p15.32	A marker when present in CSCs exhibits self-renewal potential and tumor formation.	[86]

## Data Availability

The data used to support the findings of this study are available from the corresponding authors upon request.
